# Tumoral calcinosis: Two case reports and literature review

**DOI:** 10.1097/MD.0000000000042541

**Published:** 2025-09-12

**Authors:** Xinyu Li, Jiake Chen, Zhuolin Liu, Huifen Hao, Yuanyuan Zhang

**Affiliations:** a Imaging Center, The First Affiliated Hospital, College of Clinical Medicine of Henan University of Science and Technology, Luoyang, China; b Imaging Center, Zhenggu Hospital of Luoyang, Henan Province, China.

**Keywords:** imaging features, MRI, tumoral calcinosis

## Abstract

**Rationale:**

Tumoral calcinosis is a rare, benign disorder of extraosseous calcification. Due to its rarity, it is frequently misdiagnosed as a tumor-related disease in clinical practice.

**Patient concerns:**

Both of the patients we have mentioned in this study have pathological calcification characteristics. The common denominator of these 2 cases is that the lesions are concentrated in the major joints, such as the shoulder and hip joints.

**Diagnoses:**

After undergoing imaging and related laboratory tests, both patients were diagnosed with tumoral calcinosis.

**Interventions:**

The patient had the lesion removed through surgery.

**Outcomes:**

After the surgery, the patient’s symptoms were greatly improved, and the pain was significantly reduced.

**Lessons:**

After analyzing and summarizing these cases and reviewing relevant imaging literature, we learned that the characteristic imaging manifestations of neocalcinosis have made great progress and effect on clinical diagnosis and later treatment. This knowledge can significantly aid in clinical diagnosis and treatment, thereby improving diagnostic rates and reducing instances of misdiagnosis.

## 1. Introduction

Tumoral calcinosis (TC) is a pathological entity characterized by the presence of large calcification lesion around the soft tissue.^[1–3]^ Given the lack of clear clinical symptoms and characteristic imaging findings, the disease is often mistaken for a tumor disease, resulting in diagnostic challenges and potential misdiagnosis.^[4–6]^

## 2. Case presentation

In this report, we describe 2 cases featuring substantial calcific lesions with tumoral calcifications. One case is a 44-year-old female with no history of kidney disease. Six months ago, a 6 cm diameter mass was found on the left hip of the patient. It was soft in texture, poor in motion, accompanied by swelling, without pain, and did not affect the lower limb activities and daily life. Physical examination found that the patient had normal physiological curvature and motion of each spine, and an irregular mass of about 6 cm × 6 cm in size could strike the left hip without tenderness, unclear boundary with the surrounding area, and poor motion. A series of laboratory tests on the patient showed no significant abnormalities. Computed tomography imaging showed a lobulated calcified mass with well-defined boundaries in the soft tissue surrounding the joint.^[7]^ It was subsequently surgically resected and pathologically confirmed as TC.

The other case was a male patient with a history of kidney disease.^[8]^ On October 5, 2022, the patient went to the orthopedic department for reexamination due to mass in the left shoulder and hip. The patient had a left shoulder mass 2 years ago and a left hip mass 1 year ago, and the mass was gradually increasing. The patient had chronic renal failure for 8 years and had been on regular local dialysis 3 times a week.^[9]^ He had recently developed symptoms such as anuria. In addition, he also had hypertension which was manageable with oral medication. After clinical specialist examination, it was found that the left shoulder and hip masses were poorly defined, tenderness was positive, and joint mobility was limited.

**Figure 1.**
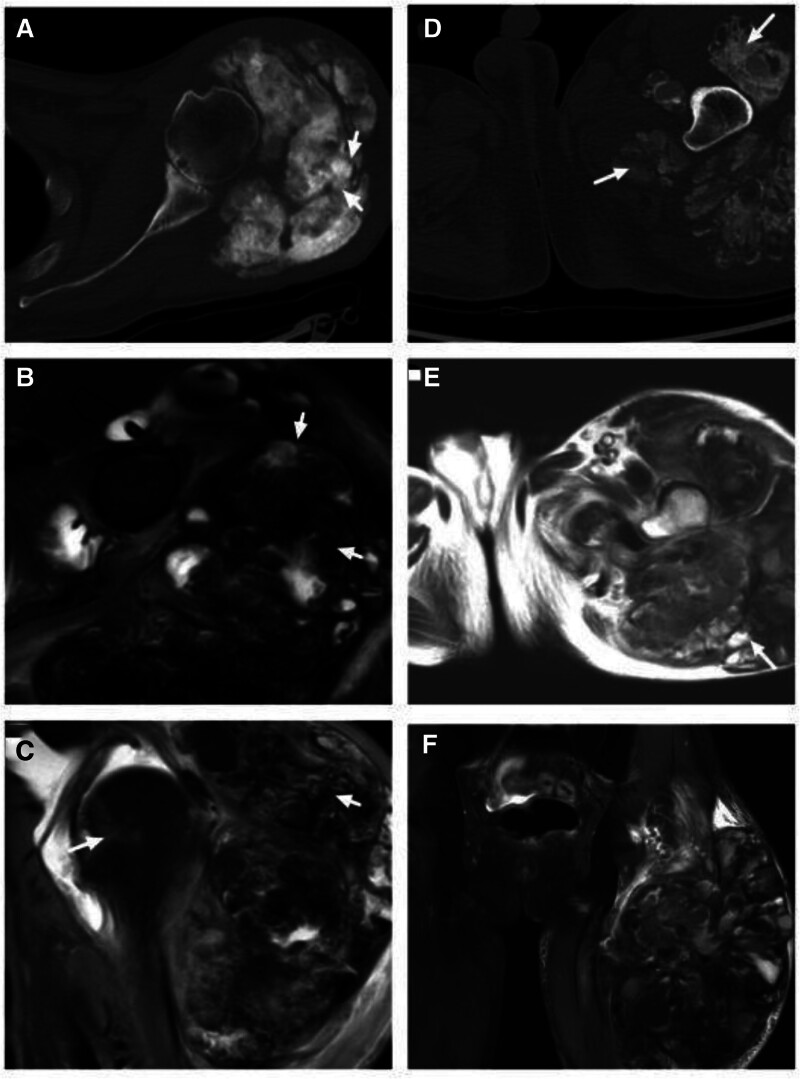
(A) CT showed thickening of soft tissue around the left shoulder joint, with clumpy lobed soft tissue shadows (white arrow). (B) MRI showed mixed signals around the shoulder joint, with cystic abnormalities mixed with low/high/slightly high T2 signals (white arrow) in the inner part. (C) Coronal MRI showing fluid accumulation in the left shoulder joint with bone marrow edema (white arrow). (D) CT showed mass mixed with high-density tissue around the left hip joint (white arrow). (E) MRI showed abnormal mixed signals in soft tissue masses around the left hip joint, partially sass-like (white arrow). (F) Abnormal cystic mixed signals were observed in coronal position. CT = computed tomography, MRI = magnetic resonance imaging.

Computed tomography imaging showed that there were clumps of calcified mass in the soft tissues around the shoulder joint and hip joint, with uneven internal density and unclear boundary. Most of the calcification foci were seen in the soft tissue of the left hip joint, local bone destruction was seen near the upper femur, and multiple blood vessels were seen in the enhanced foci (Fig. 1). Magnetic resonance imaging (MRI) showed mixed signal mass in the soft tissue around the joint, mixed under-uniform signal on both T1-weighted imaging (T1WI) and T2-weighted imaging (T2WI), and local bone destruction in the upper femur around the lesion.

**Figure 2.**
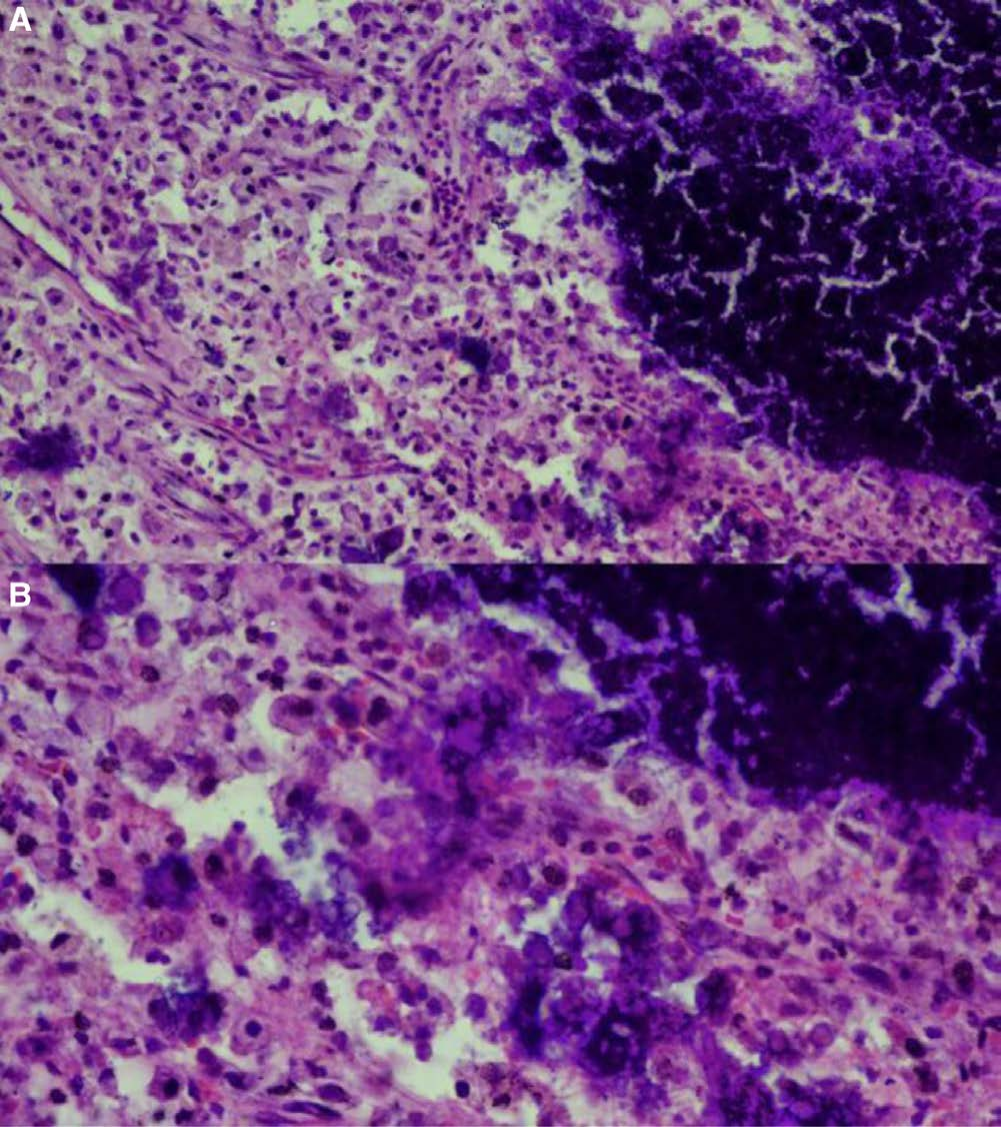
Calcified cells are aggregated, separated by fibers, surrounded by a large number of giant cells and inflammatory cells.

Laboratory tests in this patient included: creatinine increase of 620 µmol/L (normal: 40–110 µmol/L), urea increase of 30.6 µmol/L (normal: 1.7–8.3 µmol/L), potassium increase of 434 µmol/L (normal: 160–428 µmol/L), sodium decreased by 136.8 µmol/L (normal: 137–147µmol/L), and serum calcium levels were normal.^[4,7,10–13]^ After medical treatment according to the patient’s physical condition, surgical measures were taken to remove the mass, which was confirmed by pathology. The pathological findings of this patient are shown in Figure 2.

## 3. Discussion

Tumoral calcareous deposition is relatively uncommon in clinical practice compared with tumors. The most common locations of TC are the hip and shoulder. From these case reports, it can be concluded that the age range of this disease is relatively broad, with onset potential ranging from 5 to 58 years old.^[2]^ There is no obvious characteristic of the onset gender. It has been reported that most of the patients with TC have a history of renal failure and the treatment of dialysis. It can be considered as one of the complications of renal failure. Among the 2 patients discussed, one is associated with renal disease, and another patient has no history of renal disease. Other common causes include hyperparathyroidism and sarcoidosis.^[14]^

There are many conditions with similar imaging findings, such as osteomyositis and soft tissue chondroma.^[14,15]^ Osseous myositis is characterized by ectopic bone formation in soft tissue. MRI showed T1WI signal and T2WI signal to high signal.^[16]^ Soft tissue osteochondroma can occur in all parts of the soft tissue, but is more likely to occur in adults, especially in the hands and feet. MRI showed low signal on T1WI, high signal on T2WI, and unclear posterior margin and tendon.^[17]^ There are significant radiographic differences between these 2 diseases and TC. In addition to imaging, the diagnosis of TC can be based on laboratory values of serum calcium, phosphorus, parathyroid hormone, and patient history.^[18]^

In addition to the differences in imaging and clinical manifestations mentioned above, TC also differs from other diseases in terms of skin manifestations at the site of onset. In patients with dermatomyositis, purplish-red macules and calcification can be seen on the extensor side of the finger joints, and they may also have muscle weakness.^[19]^ Laboratory tests show elevated muscle enzymes, positive anti-Mi-2 antibodies, and no tumor cells. Soft tissue chondromas often occur at the proximal joints of the fingers, presenting as solitary, slow-growing, painless nodules that are hard like cartilage, with normal overlying skin and occasional local venous exposure, and usually no tenderness.^[16]^ In TC patients, large calcified masses are often seen beside the large joints, which can rupture and discharge white, paste-like or toothpaste-like calcified substances. This is a characteristic manifestation of TC and also a major clinical differentiation point.

According to the related literature, uremic TC occurs in patients with end-stage renal failure and is linked to elevated serum calcium phosphate production, often associated with secondary hyperparathyroidism.^[2,20]^ We presented a patient with tumor calcium deposits and chronic kidney disease. It has been reported that the increase of calcium phosphate production is the main factor leading to tumorigenic calcinosis.^[16]^ The abnormal calcium and phosphorus levels can be found by observing the related laboratory indexes, which can be helpful for radiographic diagnosis. Tumorous calcinosis rarely occurs in the spine. In addition, thoracic lesions are less common than cervical and lumbar lesions.^[17]^ This study aims to summarize the characteristics of the lesions through their imaging manifestations, which can enhance clinical diagnosis and facilitate the development of rational treatment plans.

**Figure 3.**
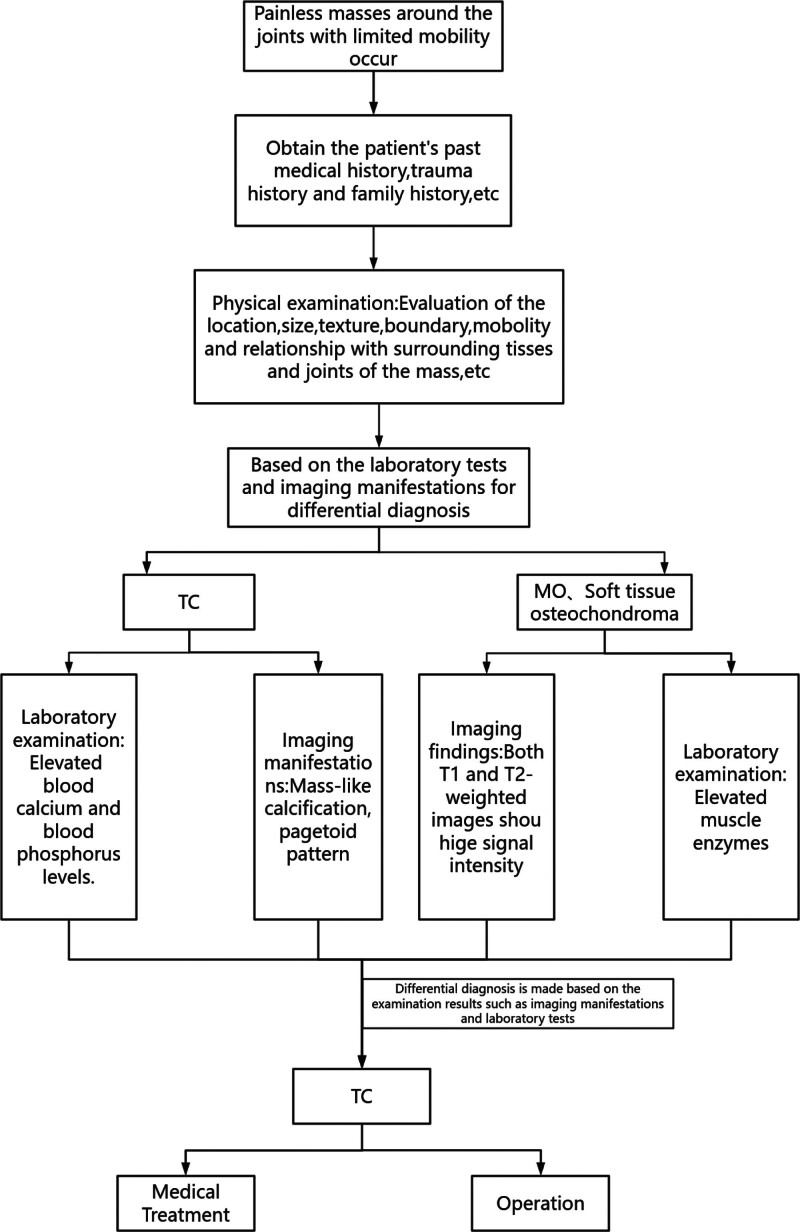
Decision-making flowchart. MO = osteomyositis, TC = tumoral calcinosis.

In our retrospective analysis of 2 patients, it was found that due to the prolonged course of the disease, severe symptoms, impact on daily life, and serious joint and bone damage involved in the lesions, the patient’s joint mobility was limited, which could not be managed conservatively, and then further surgical treatment was performed to remove the lesions to alleviate the pain of the patient. However, the existing literature indicates that the therapeutic effects of medical intervention often produce better therapeutic effects than surgical methods. Early detection and endocrine therapy can reduce pain and treatment costs. To sum up, in order to facilitate a better diagnosis of TC, the basic flowchart is shown in Figure 3.

## 4. Conclusion

Although TC is somewhat rare compared to neoplastic disease, retrospective analysis of cases and review of relevant literature suggest that calcinosis is more common in tumors, and when we see large deposits in large joints and nephropathy in patients with nephropathy, we can consider the possibility of TC in a variety of ways.^[6]^ Our goal is to summarize the imaging features of TC to better improve diagnosis rates, reduce the likelihood of misdiagnosis, promote the use of well-established medical treatment options to prioritize the disease, reduce the likelihood of surgery, and minimize risk. This approach promises to effectively address symptoms, reduce pain, and steer patients toward a path to recovery that does not require surgery.

## Author contributions

**Data curation:** Xinyu Li.

**Investigation:** Zhuolin Liu.

**Resources:** Yuanyuan Zhang.

**Validation:** Huifen Hao.

**Writing – original draft:** Jiake Chen.

**Writing – review & editing:** Xinyu Li.
